# In Silico Insights Reveal Fibronectin 1 as a Theranostic Marker in Gastric Cancer

**DOI:** 10.3390/ijms252011113

**Published:** 2024-10-16

**Authors:** Tatiana Millapán, Álvaro Gutiérrez, Krisnna Rosas, Kurt Buchegger, Carmen Gloria Ili, Priscilla Brebi

**Affiliations:** 1 Laboratory of Integrative Biology (LIBi), Centro de Excelencia en Medicina Traslacional (CEMT), Scientific and Technological Bioresource Nucleus (BIOREN), Universidad de La Frontera, Temuco 4810296, Chile; t.millapan01@ufromail.cl (T.M.);; 2 Millennium Institute on Immunology and Immunotherapy, Santiago 8331150, Chile; 3 Doctoral Program in Sciences with a Specialization in Applied Cellular and Molecular Biology, Universidad de La Frontera, Temuco 4810296, Chile; 4 Biotechnology Engineering Program, Universidad de La Frontera, Temuco 4810296, Chile; 5 BMRC, Biomedical Research Consortium, Santiago 8331150, Chile; 6 Department of Basic Sciences, Faculty of Medicine, Universidad de La Frontera, Temuco 4810296, Chile

**Keywords:** gastric cancer, theranostic markers, gene ontology, *FN1* gene

## Abstract

Gastric cancer (GC) is a complex and highly variable disease, ranking among the top five cancers diagnosed globally, and a leading cause of cancer-related deaths. Emerging from stomach lining cells amid chronic inflammation, it often advances to preneoplastic stages. Late-stage diagnoses and treatment challenges highlight the critical need for early detection and innovative biomarkers, motivating this study’s focus on identifying theranostic markers through gene ontology analysis. By exploring deregulated biological processes, this study aims to uncover insights into cancer progression and associated markers, potentially identifying novel theranostic candidates in GC. Using public data from The Human Protein Atlas, this study pinpointed 299 prognostic genes, delineating 171 with unfavorable prognosis and 128 with favorable prognosis. Functional enrichment and protein–protein interaction analyses, supported by RNAseq results and conducted via Metascape and Cytoscape, highlighted five genes (*vWF*, *FN1*, *THBS1*, *PCDH7*, and *F5*) with promising theranostic potential. Notably, *FN1* and *THBS1* exhibited significant promise, with *FN1* showing a 370% expression increase in cancerous tissue, and it is possible that *FN1* can also indicate the stratification status in GC. While further validation is essential, these findings provide new insights into molecular alterations in GC and potential avenues for clinical application of theranostic markers.

## 1. Introduction

In 2022, gastric cancer (GC) was responsible for approximately 660,000 deaths, ranking as the fifth leading cause of cancer mortality worldwide [1]. In Chile, the incidence rate of this cancer was estimated at 34.1 per 100,000 inhabitants, making it the leading cause of cancer-related deaths and contributing to 12.4% of total mortality [2]. GC originates from chronic inflammation of the gastric mucosa and progresses through preneoplastic stages [3]. Gastric adenocarcinoma accounts for approximately 90% of histological analysis diagnoses [4], with major risk factors including environmental, lifestyle, and genetic factors, among which *Helicobacter pylori* infection is a significant contributor [3,4].

Late detection, rapid progression, and lack of early symptoms contribute to diagnosis typically occurring at advanced stages, limiting treatment options and chemotherapy efficacy. Challenges such as drug resistance, absence of screening programs, and treatment costs further contribute to high mortality rates. Prioritizing primary prevention, early detection, optimal treatment, and comprehensive follow-up is crucial to improve prognosis and reduce mortality [5,6].

Advancements in the treatment of severe diseases require more precise therapeutic options to improve efficacy and reduce side effects. Despite progress in technologies such as genomics for genetic profiling, specificity in diagnosis and treatment remains a challenge. Hence, theranostics emerges as an innovative approach integrating diagnosis and therapy, facilitating precise dosing, early detection of adverse effects, and continuous treatment monitoring [7,8,9,10,11].

In this context, bioinformatics research underscores the significance of dysregulated biological processes in cancer progression. Integrating multi-omics data through high-throughput techniques captures metabolic signatures and regulatory genes involved in biosynthetic pathways, enabling comprehensive identification of endogenous metabolites and deeper insights into cancer progression. This aids in identifying activated metabolic pathways and potential therapeutic targets [12], crucial for theranostics to pinpoint cancer development markers.

Biomarkers are pivotal in theranostics, serving as quantifiable indicators of cellular alterations that elucidate physiological or pathological processes, establishing associations between etiology and disease progression [13]. They play critical roles in predicting survival, facilitating early diagnosis, and monitoring treatment [14].

This study hypothesizes that alterations in metabolic pathways significantly affect clinical outcomes during gastric cancer progression. It posits that these metabolic changes, which can lead to both favorable and unfavorable outcomes, may serve as potential biomarkers critical for the theranostic management of GC. Consequently, this study aims to analyze transcriptomic and protein databases to identify novel theranostic biomarkers for gastric cancer.

## 2. Results

### 2.1. Enrichment Analysis Highlights Unfavorable and Favorable Theranostic Genes

**Figure 1:**
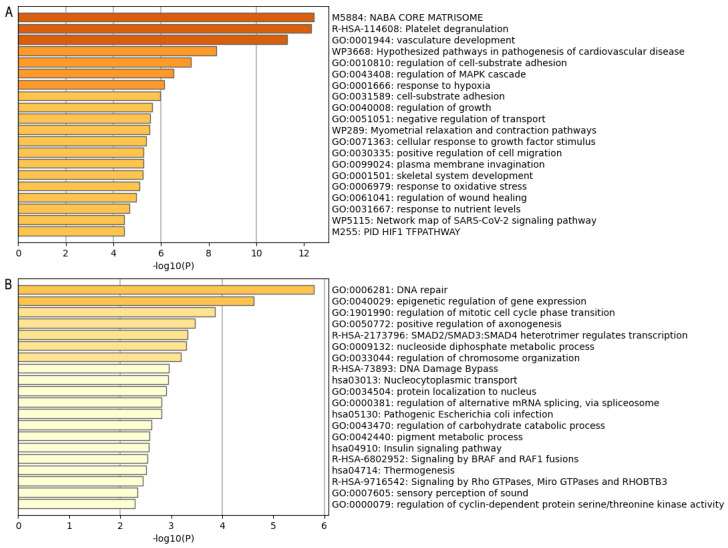
Gene ontology analysis of GC-associated genes in Metascape. (**A**) Bar chart of enriched terms correlated with the 171 genes associated with poor prognosis in GC. (**B**) Bar chart of enriched terms correlated to the 128 genes associated with good prognosis. Each term is sorted according to its *p*-value significance.

**Figure 2:**
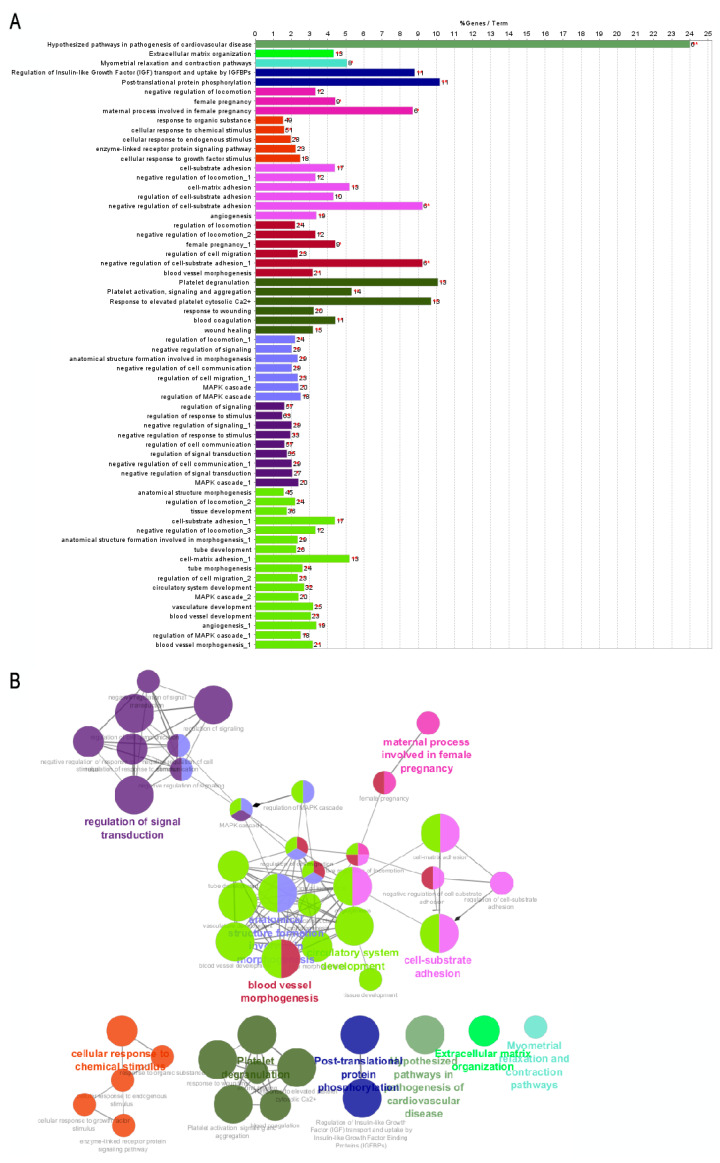
Cytoscape analysis of genes related to poor prognosis in GC. (**A**) Group A’s functional enrichment. The bars represent the percentage of genes involved in each enriched term, along with the *p*-value indicating the statistical significance of the enrichment. (**B**) Gene set network showing the relationships between genes associated with the enriched pathways. The highlighted groups demonstrate how these enriched pathways are interconnected. The asterisks in the bar chart indicate the level of statistical significance of the enriched pathways, where one asterisk (*) represents a *p*-value < 0.05 and two asterisks (**) indicate a *p*-value < 0.01.

Enrichment analysis identifies both unfavorable and favorable theranostic genes. In Group A (poor prognosis-related genes), the bar graph of enriched terms (Figure 1A) is associated with the networks of enriched terms obtained in Metascape; it can be correlated with the graph (Figure 2A) and the gene set network obtained in Cytoscape (Figure 2B). This overlapping information demonstrates significant enrichment in four common pathways: (I) Central Matriosome NABA, which consists of approximately 300 genes, encodes key proteins crucial for the architectural organization of the extracellular matrix (ECM). These genes significantly influence tumor dynamics, contributing to tumor progression and cell invasion [15]. (II) Platelet degranulation, as described by Goubran et al. [16], indicates that platelets, induced by tumor cells, promote tumor thrombosis and proliferation by releasing growth factors. This process facilitates metastasis by aiding in the migration and extravasation of cancer cells. (III) The development of vasculature emerges as a critical pathway in cancer development, facilitating the formation of a vascular network that supplies the tumor with essential blood flow to sustain its rapid growth and development [17]. (IV) Hypothesized pathways in the pathogenesis of cardiovascular disease highlight the role of shared inflammatory processes in both cardiovascular diseases and cancer. Modulating these inflammatory pathways is a crucial component of cancer treatment, as inflammation can alter the tumor microenvironment and promote tumorigenesis. Additionally, the utility of these pathways in reducing or, in extreme cases, increasing the risk of cardiovascular events has been explored [18].

**Figure 3:**
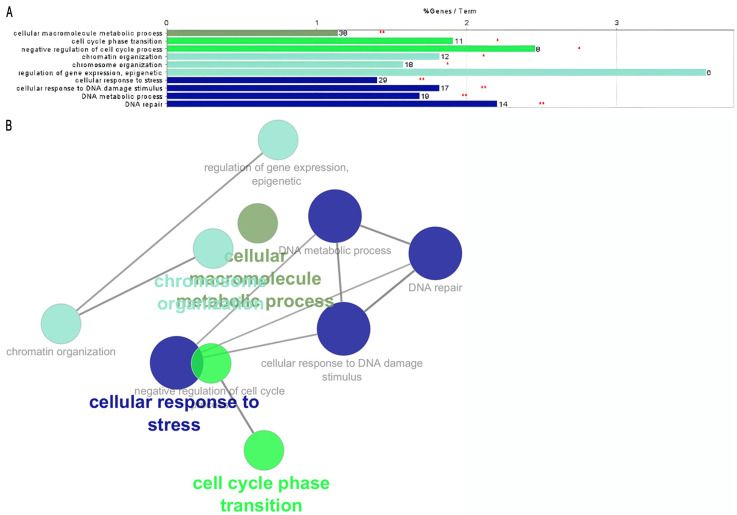
Cytoscape analysis of genes related to good prognosis in GC. (**A**) Group B’s functional enrichment was obtained from Cytoscape, highlighting key biological processes linked to favorable outcomes. (**B**) Gene set network showing the interconnectedness of genes, related to good prognosis, within the enriched terms with the enriched pathways. The asterisks in the bar chart indicate the level of statistical significance of the enriched pathways, where one asterisk (*) represents a *p*-value < 0.05 and two asterisks (**) indicate a *p*-value < 0.01.

Group B’s data, derived from both Metascape (Figure 1B) and Cytoscape (Figure 3A,B), reveal significant enrichment in two pathways: DNA repair and epigenetic regulation of gene expression. Jinjia et al. [19] reported that the expression of DNA repair-associated genes in GC samples indicates that DNA damage repair is a prevalent event in GC tumorigenesis and progression. Their findings suggest that the expression levels of these genes may serve as indicators of the intrinsic characteristics of GC, potentially acting as sensitive and specific prognostic predictors. Moreover, they observed that lower activity of DNA repair genes in cancer samples correlates with poorer prognosis.

Epigenetic regulation of gene expression constitutes the second enriched pathway. Epigenetic alterations, which are heritable changes that regulate gene expression without altering the DNA sequence, play a crucial role in cancer development [20]. These alterations, including histone modification, non-coding RNA modulation, and DNA methylation, significantly influence neoplastic development [21]. They regulate gene expression, affecting processes from the activation of oncogenes to the repression of tumor suppressor genes [22]. Thus, understanding these pathways provides valuable insights into the molecular mechanisms underpinning GC and offers potential targets for therapeutic intervention.

### 2.2. GC RNA-Seq Analysis from TCGA/GTEx

To validate some of the biomarkers found in different datasets, we performed a detailed RNA-sequencing (RNAseq) analysis using information from The Cancer Genome Atlas (TCGA-GC) and Genotype-Tissue Expression (GTEx) databases. The aim was to explore the expression patterns of 299 possible biomarkers across different cancer stages and a full set of genes, in the hope of being able to verify their roles as potential biomarkers.

Our RNAseq analysis revealed consistent patterns of dysregulation for these biomarkers across all cancer stages, from Stage I to Stage IV, highlighting three of them: fibronectin 1 (*FN1*), coagulation factor V (*F5*), and thrombospondin 1 (*THBS1*). *FN1* is a macromolecular adhesive glycoprotein and a major component of the extracellular matrix (ECM), involved in physiological and pathological processes. It is secreted by cells such as fibroblasts, chondrocytes, myocytes, and synovial and tumor cells, and is involved in cell adhesion, growth, differentiation, and migration, as well as wound healing, embryonic development, host defense, blood coagulation, and metastasis [23]. By binding to cell receptors and forming fibronectin–fibronectin complexes, *FN1* mediates communication between stromal and tumor cells, promoting angiogenesis, proliferation, and tumor metastasis, as well as interference with immune function and resistance to chemotherapy [24,25]. Factor V (*F5*) is a circulating high-molecular-weight (330 kDa) pro-cofactor involved in the blood coagulation cascade. In malignant tumors, its activation is associated with an increased risk of invasion and metastasis, resulting in a worse prognosis [26]; overexpression of *F5* in GC could contribute to poor prognosis by promoting cell migration [27]. *THBS1* is a multifunctional glycoprotein that acts as an inhibitor of angiogenesis and participates in tumor progression, where its overexpression is associated with tumor differentiation [28]. In GC, *THBS1* could have a proangiogenic effect, and its elevated expression correlates with tumor growth and lymph node metastasis [29].

**Figure 4:**
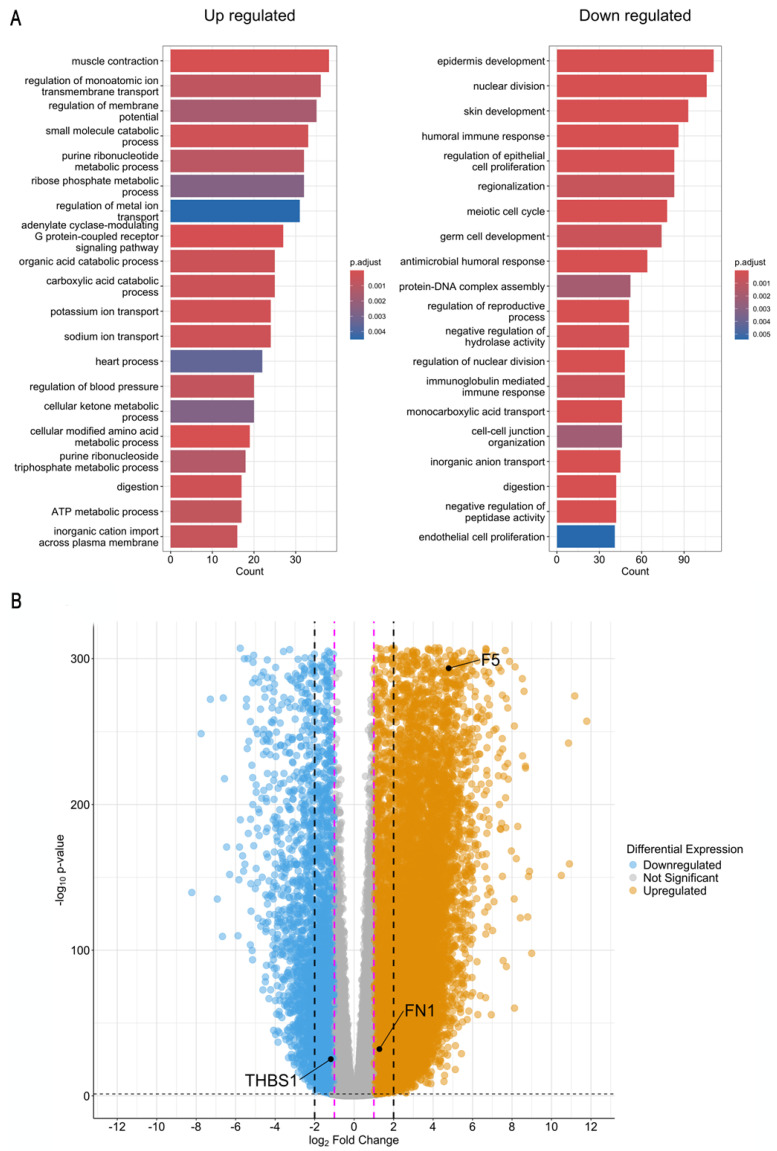
Biological process and highlighted biomarkers from TCGA transcriptional analysis. (**A**) Biological process dysregulation obtained from RNA seq analysis. All results are ordered by counts (number of genes in each pathway). (**B**) Volcano plot depicting differentially expressed genes in GC samples. Orange dots represent genes expressed at higher levels in GC samples while light-blue dots represent genes with lower expression levels in GC samples. The Y-axis denotes −log_10_ *p*-values while the X-axis shows log_2_ fold change values. Magenta and black dashed lines correspond to the range of −1 to 1 and −2 to 2 log_2_ fold change values.

Both *FN1* and *F5* were found to be positively regulated in cancer samples, with this trend observed uniformly across each stage. Specifically, *F5* exhibited a significant increase in expression, with a fold change of 4.4 across all stages, indicating a substantial upregulation. This increase in *F5* expression, which is crucial for an essential component of the coagulation cascade, implies alterations in coagulation pathways throughout the progression of cancer [27]. Similarly, *FN1* showed a slightly significant increase in its expression, with a fold change of 1.2 across all stages. Although *FN1* plays a role in the wound healing process, cancer and wound healing share common cellular and molecular pathways that operate in a delicate balance to maintain tissue homeostasis. However, when these mechanisms are deregulated, they can drive tumor progression [30]. In this context, the consistent upregulation of *FN1* affects cellular adhesion and extracellular matrix organization, suggesting a disruption of these processes as the disease progresses [31] (Figure 4).

In contrast, *THBS1* was consistently downregulated across all cancer stages. This gene exhibited a decrease in expression, with a fold change of −1.2 throughout the stages examined. *THBS1* is known for its involvement in angiogenesis and modulation of the extracellular matrix, reinforcing its role in promoting tumor growth and angiogenesis [32].

The uniform dysregulation of *FN1*, *F5*, and *THBS1* across all cancer stages highlights these genes as possible biomarkers and their potential utility in cancer diagnosis and prognosis. The consistent positive dysregulation of *FN1* and *F5*, along with the negative dysregulation of *THBS1*, suggests that these biomarkers are not only relevant for distinguishing cancerous tissues from normal ones but also for maintaining their expression patterns throughout different stages of the disease.

### 2.3. Proteomic Expression

The results obtained in both Metascape and Cytoscape reveal the recurrent presence of five genes related to Group A (Table 1), which are not only associated with enriched processes but also show significant interactions in the protein–protein network. PPI reveals some densely connected proteins by showing protein complexes such as those formed by *vWF*, *THBS1*, and *FN1*, a highly interconnected network that simultaneously presents genes in common in the two programs. These findings position *vWF*, *FN1*, *THBS1*, *PCDH7*, and *F5* as genes of special interest for this research. As for Group B, no overlapping genes are present between the enriched processes and proteins expressed in Metascape and the results obtained in Cytoscape.

### 2.4. Selected Genes as Biomarkers

**Figure 5:**
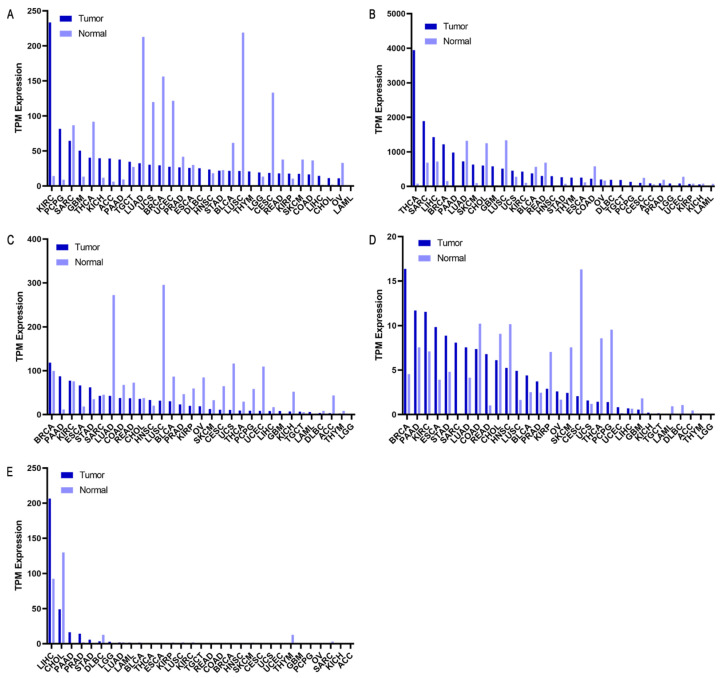
Expression profiles of potential biomarker genes across different cancer types. Each graph displays the expression levels (TPM) of (**A**) *vWF*, (**B**) *FN1*, (**C**) *THBS1*, (**D**) *PCDH7*, and (**E**) *F5* in both tumor and normal tissues. For each cancer type, two bars are presented, one for tumor tissue and one for normal tissue, allowing for direct comparison. The incidence of GC is related to STAD (stomach adenocarcinoma) in all the graphs. Data were obtained from GEPIA. Abbreviations: ACC: adrenocortical carcinoma, BLCA: bladder urothelial carcinoma, BRCA: invasive breast carcinoma, CESC: cervical squamous cell carcinoma and endocervical adenocarcinoma, CHOL: cholangiocarcinoma, COAD: colon adenocarcinoma, DLBC: diffuse large B-cell lymphoma, ESCA: esophageal carcinoma, GBM: glioblastoma multiforme, HNSC: head and neck squamous cell carcinoma, KICH: kidney chromophobe, KIRC: kidney renal clear cell carcinoma, KIRP: kidney renal papillary cell carcinoma, LAML: acute myeloid leukemia, LGG: lower grade glioma, LIHC: liver hepatocellular carcinoma, LUAD: lung adenocarcinoma, LUSC: lung squamous cell carcinoma, OV: ovarian serous cystadenocarcinoma, PAAD: pancreatic adenocarcinoma, PCPG: pheochromocytoma and paraganglioma, PRAD: prostate adenocarcinoma, READ: rectum adenocarcinoma, SARC: sarcoma, SKCM: skin cutaneous melanoma, STAD: stomach adenocarcinoma, TGCT: testicular germ cell tumor, THCA: thyroid carcinoma, THYM: thymoma, UCEC: uterine corpus endometrial carcinoma, UCS: uterine carcinosarcoma, UVM: uveal melanoma.

The results related to *vWF* selective expression in GC (Figure 5A) indicate that its expression level in cancer tissue is comparable to normal tissue (expression of 21.92 and 22.77 TPM, respectively). Its expression in normal gastric tissue is not completely selective, as it is significantly present in tissues that compose delicate organs such as the brain and heart. The interaction of *vWF* with normal stomach cell lines does not show specific staining to differentiate it from tumor cells. Furthermore, the interaction of *vWF* with cancer cells has not been described, which precludes the assumption that this marker can be optimally expressed on the cell membrane in the presence of GC. Despite the above, the Kaplan–Meier plot shows that low *vWF* expression in patients with GC is associated with better survival. Patients with low expression have a higher probability of survival compared to those with high expression, with a significant difference (*p* = 0.00023). Additionally, PPI data suggest that this marker might function more effectively in conjunction with other markers rather than as an individual biomarker (enriched protein–protein interaction groups are visualized in Figure S1 of the Supplementary Materials).

Figure 5B shows an elevated *FN1* expression during the development of GC, suggesting that it could be a promising theranostic marker. However, *FN1* is significantly expressed in a wide diversity of healthy tissues, making it difficult to use for effective site-directed therapy. Furthermore, *FN1* does not exhibit high enrichment in the stomach compared to other internal tissues, limiting its efficacy as a specific marker for GC.

According to Li et al. [24], *FN1* is primarily expressed in gastric cancer tissues, with low or no expression in adjacent normal tissues, indicating that it meets the criterion of being a protein expressed during histological progression and presenting abundant levels in neoplastic lesions, where positive staining is evident on the membrane of cancerous tissue cells. Despite presenting positive labeling in cancer tissue and negative in normal tissue, as occurs with *vWF*, *FN1* shows an increase in the plasma of patients with GC, but its expression is not direct on the surface of the cancer cells, since the protein is present in both the membranous and cytoplasmic regions. Thus, although it meets the condition of being present in the membrane of cancer cells in the presence of GC and not in normal tissue, the simultaneous presence of labeling in the cytoplasm limits its suitability to meet this specific criterion.

Of the five genes analyzed, *FN1* presents the greatest difference in expression between normal and tumor tissue, with values of 72.03 TPM in normal tissue and 266.48 TPM in tumor tissue. This difference, almost four times greater in tumor tissue, makes it a potential diagnostic marker. The marked overexpression in cancer suggests that *FN1* could be more easily detectable, which would facilitate its use in both diagnosis and early detection of tumor tissue. Although *FN1*’s survival plot also suggests its viability as a marker and shows a high expression in GC, its expression is even higher in other pathologies.

*THBS1* expression in various types of cancer reveals a predominance in invasive breast carcinoma, relegating its expression in gastric cancer (STAD) to fifth place (Figure 5C), with an expression level of 62.33 TPM, almost double its expression in normal tissue (35.21 TPM). On the other hand, *THBS1* expression in healthy tissues stands out in blood and immune cells, which could complicate the feasibility of a specific targeted therapy.

Like *FN1*, *THBS1* would appear to be an interesting marker when looking at the survival plot, but simultaneous staining, obtained by immunohistochemical data from The Human Protein Atlas (THPA), indicates that *FN1* shows elevated plasma levels in GC patients in the membrane and cytoplasm, which limits its suitability as a theranostic marker according to the criteria proposed in the research.

The expression of *PCDH7* in gastric adenocarcinoma recorded a level of 7.55 TPM (Figure 5D). In normal tissues and cell lines, the available information is limited, with profiling present mainly in the nervous system and some musculoskeletal tissues such as the heart. Negative staining is observed in normal tissue and predominantly on the cell membrane in GC, although it is also detected in the cytoplasm of cancer cells in some cases. The survival plot associated with *PCDH7* shows a lower separation between survival lines, suggesting a lesser involvement of this gene in survival.

*F5* exhibits an expression of 5.89 in gastric adenocarcinoma (Figure 5E), surpassing *PCDH7* in terms of the lowest expression in GC. Similar to *PCDH7*, information regarding *F5* expression in normal cell lines is incomplete, with no specifications found regarding its expression in the stomach. However, data from GeneCards show its high expression on blood and immune system tissues, aligning with its role in blood coagulation. The Kaplan–Meier plot from *F5* does not show as notable a difference as other previously presented plots.

For each gene analyzed, the supplementary figures (Figures S2, S3, S4, S5, and S6) include detailed information on protein expression, tissue staining patterns, and Kaplan–Meier survival curves.

## 3. Discussion

The challenges in gastric cancer treatment have driven the development of approaches that integrate both diagnosis and therapy. Theranostic agents have emerged as promising tools, uniting these two areas into a cohesive process that integrates diagnosis, therapy, and monitoring of therapeutic response into a single integrated process to enable personalized treatment strategies that can potentially improve patient outcomes [33]. In GC, various diagnostic markers, such as CEA, CA19-9, CA72-4, HLA-G, IL-6, and PD-1 [34,35] have been proposed, and therapeutic targets like HER2, primarily used in advanced stages, have been tested as chemotherapeutic treatments [34,36]. Circulating molecules with diagnostic potential, including miRNA, lncRNA, and circRNA [37], offer promising new strategies for early GC detection. However, there is still no consensus on a single target that fulfills both diagnostic and therapeutic roles, highlighting the need to identify true theranostic agents to enhance the clinical management of GC. This study investigates a set of genes associated with dysregulated pathways to assess their potential in improving the diagnosis and treatment of GC.

Most cancer-causing mutations affect the DNA sequence (genetic mutations), while others are dynamic, heritable changes independent of the DNA sequence (epigenetic mutations). DNA mutations can be irreversible, such as point mutations, chromosomal rearrangements, deletions, and duplications, or reversible, like epigenetic modifications that alter methylation patterns and histone post-translational modifications [38]. In contrast, pathways such as central Matrisome NABA, platelet degranulation, the development of vasculature, and hypothesized pathways in the pathogenesis of cardiovascular disease—characterized by promoting a tumor-supportive microenvironment and facilitating tumor progression and metastasis—were linked to genes associated with poor prognosis. Among the enriched pathways related to poor prognosis, five theranostic markers of interest were identified as deregulated and enriched in gastric cancer, including *vWF*, *FN1*, *THBS1*, *PCDH7*, and *F5*.

The von Willebrand factor is the largest multimeric protein in human blood, crucial for platelet adhesion to the subendothelial matrix and endothelial surfaces [39]. GC cells express and secrete *vWF*, promoting cell aggregation, platelet interaction, and GC growth/metastasis, suggesting a significant role in GC growth and metastasis [40,41]. GC patients show elevated plasma *vWF* levels, and the adhesive activity of *vWF* is enhanced in GC, binding to lyophilized platelets via the GP Ib-IX-V complex and the subendothelial matrix [42]. This indicates a self-propagation process where *vWF* binds and activates platelets, releasing more hyperadhesive *vWF* stored in α-granules, contributing to cancer progression. On the other hand, equal expression of this gene in both cancerous and normal tissue indicates no differential predominance during cancer development. Its high presence in delicate tissues complicates its application as a potential theranostic marker. Although numerous staining images are available, the interaction of *vWF* with cancer cells is not well described, suggesting that it may not be optimally expressed on the cell membrane in the presence of GC. Despite the survival curve indicating its potential as a prognostic marker, its high presence in delicate tissues limits its use as a theranostic marker. However, *vWF* could still provide information about disease progression as its plasma levels and activity increase in advanced stages of GC [43].

Fibronectin 1 binds to cell surface receptors, mediating cross-talk between stromal and tumor cells, and interacting with integrin receptors to promote tumor progression by interfering with immune function or chemotherapy resistance [24]. *FN1* is upregulated in GC tissues compared to adjacent normal tissues and is associated with poor prognosis, as reported by Wang et al. [31] and Sun et al. [44]. These findings are consistent with the results of this study.

*FN1*’s interaction with transforming growth factor-beta (TGF-β) influences cell signaling and growth regulation, with TGF-β increasing the expression of cyclin inhibitors and inducing cell death through various pathways. During tumorigenesis, cells develop resistance to TGF-β’s inhibitory effects, promoting tumor progression. This dual behavior of TGF-β presents opportunities for developing antitumor drugs that specifically inhibit the tumorigenesis-promoting effects induced by TGF-β. The mRNA expression of FN1 is considered more clinically relevant than the *FN1* protein itself, with the FN1 3′-UTR promoting aggressive invasion and metastasis in GC, suggesting that it is a better pharmacological target [45,46]. The possibility of using these pathways as a possible therapeutic target opens the doors to future studies that could bring this gene closer to theranostic suitability.

Thrombospondin 1’s increased expression is associated with tumor differentiation, growth, and lymph node metastasis in gastric cancer [28,29]. Despite its simultaneous staining in the membrane and cytoplasm limiting its suitability as a theranostic marker, *THBS1* is considered a potential therapeutic target due to its role in tumor progression and immune response [47,48]. Using *THBS1* as a therapeutic target could enhance the efficacy of anticancer drugs, with studies showing that positive regulation of *THBS1* leads to activation of the PI3K-Akt signaling pathway, promoting tumor growth and resistance to therapy [49]. This underscores the potential of *THBS1* and associated pathways as targets for developing more effective treatments. Additionally, *THBS2*, related to *THBS1*, fulfills the criteria of selectivity and high expression in cancerous tissue, providing valuable information on the biochemical and morphological characteristics of the affected region.

*PCDH7*, a member of the protocadherin family, is a key membrane protein involved in cell recognition and adhesion. Its negative regulation in gastric and bladder cancer suggests a tumor suppressor role by inhibiting cell migration and invasion [50,51]. *PCDH7* could be a marker for GC theranostics due to its potential detection on the cell surface. However, its signaling pathways in GC have not been identified. In other cancers, *PCDH7* confers chemoresistance by inhibiting apoptosis and promoting anti-apoptotic protein expression and Wnt signaling, which could be targeted for GC treatment [52].

Coagulation factor V (*F5*) is crucial in the blood coagulation cascade, with its activation associated with increased invasion, metastasis, and poor prognosis in GC [26,27]. *F5* expression in GC tissues correlates with tumor grade and stage, providing information on the biochemical characteristics of pathology development [53]. Although high expression in the circulatory system complicates its targeted therapy application, *F5*’s role in regulating the Wnt and TGF-β signaling pathways suggests potential therapeutic intervention points. Studies also identify *F5* as a D-dimer-related gene, with implications for immune response and cancer survival, suggesting additional regulatory pathways in GC [26,54].

Overall, these findings highlight the complex roles of *vWF*, *FN1*, *THBS1*, *PCDH7*, and *F5* in GC progression and their potential as biomarkers and therapeutic targets. Further research is necessary to fully understand their mechanisms and develop targeted treatments.

## 4. Methods and Materials

### 4.1. Genomic Data

Public data from The Human Protein Atlas (THPA) regarding gastric cancer, which integrates transcriptomic data from The Cancer Genome Atlas (TCGA) and antibody-based protein data, were utilized in this study. Transcriptomic profiles from 354 patients were analyzed, identifying 299 genes with prognostic potential, categorized into 171 genes associated with unfavorable prognosis (Group A) and 128 with favorable prognosis (Group B).

### 4.2. Enrichment Analysis

Two bioinformatics tools were employed for enrichment analysis. The first tool was the Metascape database (version v3.5.20240901), where terms meeting enrichment criteria (enrichment factor > 1.5, minimum count > 3, and *p* < 0.01) were deemed significant, following the methodology outlined by Dang et al. [55]. Protein–protein interaction (PPI) analysis was conducted automatically using STRING, BioGrid, OmniPath, and InWeb_IM databases. Molecular complex detection (MCODE) was applied with parameters set to detect densely connected network components (degree limit = 2, node score limit = 0.2, k-core = 2, maximum depth = 100).

The second tool employed was Cytoscape (version v3.10.1) [56], utilizing the ClueGO plugin [57] for enrichment analysis. Each gene list was independently analyzed against KEGG, GO-Biological Process, REACTOME-Pathways, and WikiPathways databases. GO term fusion was applied with a significance threshold set at *p* ≤ 0.03 and *p* ≤ 0.05, focusing on specificity at levels 3 and 2. Enrichment analysis results were visually represented as a network of gene sets (nodes).

### 4.3. Theranostic Evaluation Criteria

Following Jokerst and Gambhir’s [58] criteria for theranostic probes, this study considers three out of four essential characteristics for ideal theranostic probes. The markers should (i) accumulate rapidly and selectively in affected tissue; (ii) provide information on biochemical and/or morphological characteristics of the region; and (iii) enable effective site-directed therapeutic administration. The fourth characteristic (being safe and biodegradable) is particularly relevant to nanomaterials, and safety considerations are integrated into the fulfillment of the first listed characteristic.

A primary criterion for selecting biological markers is their preferential expression on the surface of cancer cells, minimizing cytoplasmic or nuclear localization. Additionally, markers targeting non-internalizing receptors depend on their enrichment in diseased tissue, aiding in the identification of optimal tissue biomarker accumulation and selectivity based on two criteria: (i) proteins specifically expressed in the cell of origin, highly enriched in the organ/tissue, and consistently preserved during the transition from normal to neoplastic tissue; and (ii) proteins not initially expressed in the cell of origin but exhibiting abundant and conserved levels during histological progression in a significant percentage of cells within neoplastic lesions [59,60].

### 4.4. GC RNA-Seq Analysis from TCGA/GTEx

To validate gene expression and biomarker discovery, an RNAseq analysis was performed. RNAseq data for gene expression analysis were obtained from The Cancer Genome Atlas (TCGA) for gastric cancer samples (484 samples = from the project IDs of TCGA-STAD, TCGA-SARC, TCGA-ESCA, and HCMI-CMDC) and from the Genotype-Tissue Expression (GTEx) project for normal tissue controls (359 samples = from Bulk Tissue Expression, Analysis V8, version 1.1.9). Cancer patients were stratified into four groups based on cancer stages (I, II, III, and IV). Bioinformatics analysis relied solely on read counts obtained from RNAseq data. Differential gene expression between cancer and normal tissue datasets was assessed using DESeq2 v.1.36.0 [61] in R (4.2.1), with genes showing a |log2 fold change| ≥ 1 and an adjusted *p*-value < 0.05 considered significantly differentially expressed. Gene ontology (GO) and Kyoto Encyclopedia of Genes and Genomes (KEGG) enrichment analyses were performed using ClusterProfiler v.3.14.3 [62,63,64] in R (3.6.3).

### 4.5. Genomic and Proteomic Distribution and Expression of Cancer Cell Lines

Protein expression values of the genes of interest in different types of cancer and in normal tissue were obtained from GEPIA. These data have been tabulated in Prism and were used to generate data plots. The distribution and expression of the presented genes are based on information from GEPIA, using data from the TCGA Research Network, accessible via the provided link: http://gepia.cancer-pku.cn/ (accessed on 7 July 2024). For the expression of genes as potential theranostic markers linked to GC, immunohistochemistry image data available from The Human Protein Atlas were collected. Information on survival associated (IHQ) with these genes was also obtained from the same database.

### 4.6. Proteomic Expression in Normal Tissues and Cell Lines

To evaluate protein expression in normal tissues and cell lines, the GeneCards platform was used. This database provides expression profiles focused on normal human tissues, offering two sets of tissue vectors. One of these sets is based on experimental DNA array results patented by the Weizmann Institute of Science, while the other is based on in silico data extraction and the quantification of Expressed Sequence Tags (ESTs) from a selected set of tissues in Unigene clusters [65,66]. The information collected from GeneCards played a crucial role in contextualizing normal protein expression levels, providing an essential reference framework for comparison with the tumor samples analyzed in this study.

## 5. Conclusions

Gene ontology analysis in gastric cancer identified five potential theranostic markers, deregulated and enriched in GC. These markers, associated with poor prognosis, correspond to *vWF*, *FN1*, *THBS1*, *PCDH7*, and *F5*. Among them, both *FN1* and *THBS1* show remarkable characteristics for theranostic application. *THBS1* is twice as highly expressed in cancerous tissue compared to normal tissue and is linked to tumor growth in gastric cancer. On the other hand, *FN1* increases its expression in cancerous tissue by 370% compared to normal tissue. Based on the information gathered, *FN1* may also indicate stratification status in GC, due to previous investigations that have proposed it as a promising biomarker for this pathology. Unfortunately, its high expression in healthy tissue is present in many different cell lines, which would make site-directed therapy difficult. However, future research could address this drawback by exploring the possibility of using secondary structures that provide protection and/or selectivity to the protein towards sensitive tissues.

Although this study has investigated theranostic criteria for marker selection, the main limitation is the lack of an in vitro study of the expression of the selected genes that would provide more detailed information. In addition, preclinical validation is necessary to support the potential use of the selected genes as theranostic markers.

Finally, the genes identified in this study as potential theranostic markers open up new avenues of research for targeted therapy in gastric cancer. We hope that these findings will stimulate studies that address the limitations encountered and allow for safer and more effective clinical applications in the future.

## Figures and Tables

**Table 1:** Genes linked to biological processes enriched in Group A and Group B, obtained through enrichment analysis performed in Cytoscape.

Group	ID	Enriched Process	Associated Genes
A	WP:3668	Hypothesized pathways in pathogenesis of cardiovascular disease	*ANGPT2*, *CCN2*, *FBN1*, *POSTN*, *RUNX2*, *SERPINE1*
	R-HSA:8957275	Post-translational protein phosphorylation process	*ALB*, *F5*, *FBN1*, *FN1*, *GPC3*, *IGFBP1*, *MATN3*, *MFGE8*, *MXRA8*, *STC2*, *VCAN*
	R-HSA:114608	Platelet degranulation	*ALB*, *ANXA5*, *CD109*, *CD36*, *F5*, *FN1*, *MAGED2*, *MMRN1*, *PCDH7*, *PROS1*, *SERPINE1*, *THBS1*, *VWF*
B	GO:0040029	Epigenetic regulation of gene expression	*EZH2*, *KCNQ1*, *LMNB2*, *RBM15*, *SAMD1*, *SIRT6*
	GO:0010948	Negative regulation of cell cycle process	*CLSPN*, *CUL4A*, *ESPL1*, *EZH2*, *FBXO5*, *INIP*, *MEN1*, *NACC2*
	GO:0006281	DNA repair	*CHAF1A*, *CLSPN*, *CUL4A*, *FEN1*, *INIP*, *INO80B*, *MEN1*, *POLQ*, *SIRT6*, *SWSAP1*, *TAF5*, *TMEM161A*, *USP43*, *ZBTB7A*

## Data Availability

The original data used in this study were obtained from The Human Protein Atlas (THPA) and were further processed using Metascape and Cytoscape. These data are openly available at https://www.proteinatlas.org/.

## Supplementary Materials

The following supporting information can be downloaded at: https://www.mdpi.com/article/10.3390/ijms252011113/s1.

## References

[1] Bray F., Laversanne M., Sung H., Ferlay J., Siegel R.L., Soerjomataram I., Jemal A. (2024). Global cancer statistics 2022: GLOBOCAN estimates of incidence and mortality worldwide for 36 cancers in 185 countries. CA Cancer J. Clin..

[2] Ministerio de Salud (2020). Guía de Práctica Clínica Cáncer Gástrico.

[3] Bornschein J., Quante M., Fassan M., Kuipers E.J. (2020). Gastric Cancer; Epidemiology and Diagnosis. Encyclopedia of Gastroenterology.

[4] Ilic M., Ilic I. (2022). Epidemiology of stomach cancer. World J. Gastroenterol..

[5] Matsuoka T., Yashiro M. (2018). Biomarkers of gastric cancer: Current topics and future perspective. World J. Gastroenterol..

[6] Riquelme I., Ili C., Roa J.C., Brebi P. (2016). Long non-coding RNAs in gastric cancer: Mechanisms and potential applications. Oncotarget.

[7] Kojima R., Aubel D., Fussenegger M. (2015). Novel theranostic agents for next-generation personalized medicine: Small molecules, nanoparticles, and engineered mammalian cells. Curr. Opin. Chem. Biol..

[8] Lee S.T., Kulkarni H.R., Singh A., Baum R.P. (2017). Theranostics of Neuroendocrine Tumors. Visc. Med..

[9] Li X., Kim J., Yoon J., Chen X. (2017). Cancer-Associated, Stimuli-Driven, Turn on Theranostics for Multimodality Imaging and Therapy. Adv. Mater..

[10] Ryu J.H., Lee S., Son S., Kim S.H., Leary J.F., Choi K., Kwon I.C. (2014). Theranostic nanoparticles for future personalized medicine. J. Control. Release.

[11] Yu G., Jiang M., Huang F., Chen X. (2021). Supramolecular coordination complexes as diagnostic and therapeutic agents. Curr. Opin. Chem. Biol..

[12] Gao X., Guo R., Li Y., Kang G., Wu Y., Cheng J., Jia J., Wang W., Li Z., Wang A. (2021). Contribution of upregulated aminoacyl-tRNA biosynthesis to metabolic dysregulation in gastric cancer. J. Gastroenterol. Hepatol..

[13] Camacho-Sánchez M., Leandro-Vargas L.A., Mendoza-Salas M., Meza-Gutiérrez N., Montero-Zúñiga F. (2023). Biomarcadores en el diagnóstico temprano y tratamiento de cáncer. Rev. Tecnol. En Marcha.

[14] Mérida de la Torre F.J., Moreno Campoy E.E. (2019). Papel diagnóstico de los marcadores tumorales. Med. Clín. (Ed. Impr.).

[15] Socovich A.M., Naba A. (2019). The cancer matrisome: From comprehensive characterization to biomarker discovery. Semin. Cell Dev. Biol..

[16] Goubran H.A., Stakiw J., Radosevic M., Burnouf T. (2014). Platelets Effects on Tumor Growth. Semin. Oncol..

[17] Li J., Han T. (2023). Comprehensive analysis of the oncogenic roles of vascular endothelial growth factors and their receptors in stomach adenocarcinoma. Heliyon.

[18] Libby P., Kobold S. (2019). Inflammation: A common contributor to cancer, aging, and cardiovascular diseases—Expanding the concept of cardio-oncology. Cardiovasc. Res..

[19] Chang J., Wang X., Sun H., Li W., Zhang Z., Zhu X., Xu M. (2019). The use of DNA repair genes as prognostic indicators of gastric cancer. J. Cancer.

[20] Ebrahimi V., Soleimanian A., Ebrahimi T., Azargun R., Yazdani P., Eyvazi S., Tarhriz V. (2020). Epigenetic modifications in gastric cancer: Focus on DNA methylation. Gene.

[21] Sonohara F., Inokawa Y., Hayashi M., Kodera Y., Nomoto S. (2017). Epigenetic modulation associated with carcinogenesis and prognosis of human gastric cancer (Review). Oncol. Lett..

[22] Samadani A.A., Noroollahi S.E., Mansour-Ghanaei F., Rashidy-Pour A., Joukar F., Bandegi A.R. (2019). Fluctuations of epigenetic regulations in human gastric Adenocarcinoma: How does it affect?. Biomed. Pharmacother..

[23] Tossetta G., Avellini C., Licini C., Giannubilo S., Castellucci M., Marzioni D. (2016). High temperature requirement A1 and fibronectin: Two possible players in placental tissue remodelling. Eur. J. Histochem..

[24] Li J., Chen C., Chen B., Guo T. (2022). High FN1 expression correlates with gastric cancer progression. Pathol. Res. Pract..

[25] Pan H., Luo Z., Lin F., Zhang J., Xiong T., Hong Y., Sun B., Yang Y. (2024). FN1, a reliable prognostic biomarker for thyroid cancer, is associated with tumor immunity and an unfavorable prognosis. Oncol. Lett..

[26] Tang M., Yang B., Zhang C., Zhang C., Zang D., Gong L., Liu Y., Li Z., Qu X. (2021). The F5 gene predicts poor prognosis of patients with gastric cancer by promoting cell migration identified using a weighted gene co-expression network analysis. Biocell.

[27] Liu Y., Liao X.-W., Qin Y.-Z., Mo X.-W., Luo S.-S. (2020). Identification of F5 as a Prognostic Biomarker in Patients with Gastric Cancer. BioMed Res. Int..

[28] Yang X., Chen L., Mao Y., Hu Z., He M. (2020). Progressive and Prognostic Performance of an Extracellular Matrix-Receptor Interaction Signature in Gastric Cancer. Dis. Markers.

[29] Hong B.-B., Chen S.-Q., Qi Y.-L., Zhu J.-W., Lin J.-Y. (2015). Association of THBS1 rs1478605 T>C in 5′-untranslated regions with the development and progression of gastric cancer. Biomed. Rep..

[30] López-Cortés A., Abarca E., Silva L., Velastegui E., León-Sosa A., Karolys G., Cabrera F., Caicedo A. (2021). Identification of key proteins in the signaling crossroads between wound healing and cancer hallmark phenotypes. Sci. Rep..

[31] Wang H., Zhang J., Li H., Yu H., Chen S., Liu S., Zhang C., He Y. (2022). FN1 is a prognostic biomarker and correlated with immune infiltrates in gastric cancers. Front. Oncol..

[32] Eskandarion M.R., Eskandarieh S., Farahani A.S., Mahmoodzadeh H., Shahi F., Oghabian M.A., Shirkoohi R. (2024). Prediction of novel biomarkers for gastric intestinal metaplasia and gastric adenocarcinoma using bioinformatics analysis. Heliyon.

[33] Nyakale N.E., Aldous C., Gutta A.A., Khuzwayo X., Harry L., Sathekge M.M. Emerging theragnostic radionuclide applications for hepatocellular carcinoma. Front. Nucl. Med..

[34] Ye D.M., Xu G., Ma W., Li Y., Luo W., Xiao Y., Liu Y., Zhang Z. (2020). Significant function and research progress of biomarkers in gastric cancer (Review). Oncol. Lett..

[35] Repetto O., Vettori R., Steffan A., Cannizzaro R., De Re V. (2023). Circulating Proteins as Diagnostic Markers in Gastric Cancer. Int. J. Mol. Sci..

[36] Sato Y., Okamoto K., Kida Y., Mitsui Y., Kawano Y., Sogabe M., Miyamoto H., Takayama T. (2023). Overview of Chemotherapy for Gastric Cancer. J. Clin. Med..

[37] Necula L., Matei L., Dragu D., Neagu A.I., Mambet C., Nedeianu S., Bleotu C., Diaconu C.C., Chivu-Economescu M. (2019). Recent advances in gastric cancer early diagnosis. World J. Gastroenterol..

[38] Farhana A., Yusuf N., Rasheed Z. (2024). Editorial: Cancer genetics and epigenetics: Theranostic targets and mechanisms. Front. Genet..

[39] Franchini M., Frattini F., Crestani S., Bonfanti C., Lippi G. (2013). von Willebrand factor and cancer: A renewed interest. Thromb. Res..

[40] Yang A.-J., Wang M., Wang Y., Cai W., Li Q., Zhao T.-T., Zhang L.-H., Houck K., Chen X., Jin Y.-L. (2018). Cancer cell-derived von Willebrand factor enhanced metastasis of gastric adenocarcinoma. Oncogenesis.

[41] Wang C.Y., Wang M., Cai W., Zhao C.Y., Zhou Q., Zhang X.Y., Wang F.X., Zhang C.L., Dang Y., Yang A.J. (2023). Von Willebrand Factor Synergizes with Tumor-Derived Extracellular Vesicles to Promote Gastric Cancer Metastasis. bioRxiv.

[42] Cai W., Wang M., Wang C.-Y., Zhao C.-Y., Zhang X.-Y., Zhou Q., Zhao W.-J., Yang F., Zhang C.-L., Yang A.-J. (2023). Extracellular vesicles, hyperadhesive von willebrand factor, and outcomes of gastric cancer: A clinical observational study. Med. Oncol..

[43] Yang X., Sun H.-J., Li Z.-R., Zhang H., Yang W.-J., Ni B., Wu Y.-Z. (2015). Gastric cancer-associated enhancement of von Willebrand factor is regulated by vascular endothelial growth factor and related to disease severity. BMC Cancer.

[44] Sun Y., Zhao C., Ye Y., Wang Z., He Y., Li Y., Mao H. (2020). High expression of fibronectin 1 indicates poor prognosis in gastric cancer. Oncol. Lett..

[45] Seoane J., Gomis R.R. (2017). TGF-β Family Signaling in Tumor Suppression and Cancer Progression. Cold Spring Harb. Perspect. Biol..

[46] Pan S., Zhu J., Liu P., Wei Q., Zhang S., An W., Tong Y., Cheng Z., Liu F. (2023). FN1 mRNA 3′-UTR supersedes traditional fibronectin 1 in facilitating the invasion and metastasis of gastric cancer through the FN1 3′-UTR-let-7i-5p-THBS1 axis. Theranostics.

[47] Li Y., Wang J.-S., Zhang T., Wang H.-C., Li L.-P. (2020). Identification of New Therapeutic Targets for Gastric Cancer with Bioinformatics. Front. Genet..

[48] Zhang X., Huang T., Li Y., Qiu H. (2021). Upregulation of THBS1 is Related to Immunity and Chemotherapy Resistance in Gastric Cancer. Int. J. Gen. Med..

[49] Khoshdel F., Mottaghi-Dastjerdi N., Yazdani F., Salehi S., Ghorbani A., Montazeri H., Soltany-Rezaee-Rad M., Goodarzy B. (2024). CTGF, FN1, IL-6, THBS1, and WISP1 genes and PI3K-Akt signaling pathway as prognostic and therapeutic targets in gastric cancer identified by gene network modeling. Discov. Oncol..

[50] Chen H.-F., Ma R.-R., He J.-Y., Zhang H., Liu X.-L., Guo X.-Y., Gao P. (2017). Protocadherin 7 inhibits cell migration and invasion through E-cadherin in gastric cancer. Tumour Biol..

[51] Zhang Y., Lyu Y., Chen L., Cao K., Chen J., He C., Lyu X., Jiang Y., Xiang J., Liu B. (2023). Exploring the Prognosis-Related Genetic Variation in Gastric Cancer Based on mGWAS. Int. J. Mol. Sci..

[52] Zheng Z., Luan N., Tu K., Liu F., Wang J., Sun J. (2023). The roles of protocadherin-7 in colorectal cancer cells on cell proliferation and its chemoresistance. Front. Pharmacol..

[53] Guan Y., Xu B., Sui Y., Chen Z., Luan Y., Jiang Y., Wei L., Long W., Zhao S., Han L. (2022). Pan-Cancer Analysis and Validation Reveals that D-Dimer-Related Genes are Prognostic and Downregulate CD8+ T Cells via TGF-Beta Signaling in Gastric Cancer. Front. Mol. Biosci..

[54] Chiurillo M.A. (2015). Role of the Wnt/β-catenin pathway in gastric cancer: An in-depth literature review. World J. Exp. Med..

[55] Dang K., Farooq H.M.U., Dong J., Yang H., Kong Y., Wang H., Jiang S., Gao Y., Qian A. (2023). Transcriptomic and proteomic time-course analyses based on Metascape reveal mechanisms against muscle atrophy in hibernating *Spermophilus dauricus*. Comp. Biochem. Physiol. Part A Mol. Integr. Physiol..

[56] Shannon P., Markiel A., Ozier O., Baliga N.S., Wang J.T., Ramage D., Amin N., Schwikowski B., Ideker T. (2003). Cytoscape: A Software Environment for Integrated Models of Biomolecular Interaction Networks. Genome Res..

[57] Bindea G., Mlecnik B., Hackl H., Charoentong P., Tosolini M., Kirilovsky A., Fridman W.-H., Pagès F., Trajanoski Z., Galon J. (2009). ClueGO: A Cytoscape plug-in to decipher functionally grouped gene ontology and pathway annotation networks. Bioinformatics.

[58] Jokerst J.V., Gambhir S.S. (2011). Molecular Imaging with Theranostic Nanoparticles. Acc. Chem. Res..

[59] Srinivasarao M., Galliford C.V., Low P.S. (2015). Low PS. Principles in the design of ligand-targeted cancer therapeutics and imaging agents. Nat. Rev. Drug Discov..

[60] Espinoza J.A., Riquelme I., Sagredo E.A., Rosa L., García P., Bizama C., Apud-Bell M., Leal P., Weber H., Benavente F. (2018). Mucin 5B, carbonic anhydrase 9 and claudin 18 are potential theranostic markers of gallbladder carcinoma. Histopathology.

[61] Love M.I., Huber W., Anders S. (2014). Moderated estimation of fold change and dispersion for RNA-seq data with DESeq2. Genome Biol..

[62] Yu G., Wang L.-G., Han Y., He Q.-Y. (2012). clusterProfiler: An R Package for Comparing Biological Themes Among Gene Clusters. OMICS J. Integr. Biol..

[63] Xu S., Hu E., Cai Y., Xie Z., Luo X., Zhan L., Tang W., Wang Q., Liu B., Wang R. (2024). Using clusterProfiler to characterize multiomics data. Nat. Protoc..

[64] Wu T., Hu E., Xu S., Chen M., Guo P., Dai Z., Feng T., Zhou L., Tang W., Zhan L. (2021). clusterProfiler 4.0: A universal enrichment tool for interpreting omics data. Innovation.

[65] Safran M., Chalifa-Caspi V., Shmueli O., Olender T., Lapidot M., Rosen N., Shmoish M., Peter Y., Glusman G., Feldmesser E. (2003). Human Gene-Centric Databases at the Weizmann Institute of Science: GeneCards, UDB, CroW 21 and HORDE. Nucleic Acids Res..

[66] Fishilevich S., Zimmerman S., Kohn A., Stein T.I., Olender T., Kolker E., Safran M., Lancet D. (2016). Genic insights from integrated human proteomics in GeneCards. Database.

