# Supplementary material for: In Silico Insights Reveal Fibronectin 1 as a Theranostic Marker in Gastric Cancer
Source: Int J Mol Sci. 2024 Oct 16;25(20):11113. doi: 10.3390/ijms252011113 (PMC11507984; doi:10.3390/ijms252011113)
Supplement: Supplementary file 1 [file ijms-25-11113-s001.zip › Supplementary Figures.pdf]

# In Silico Insights Reveal Fibronectin 1 as a Theranostic Marker in Gastric Cancer

Tatiana Millapán[1,2], Álvaro Gutiérrez[1,2,5], Krisnna Rosas[1,2,4], Kurt Buchegger[1,2,3,6], Carmen Gloria Ili[1,2,3*], Priscilla Brebi[1,2,3*]

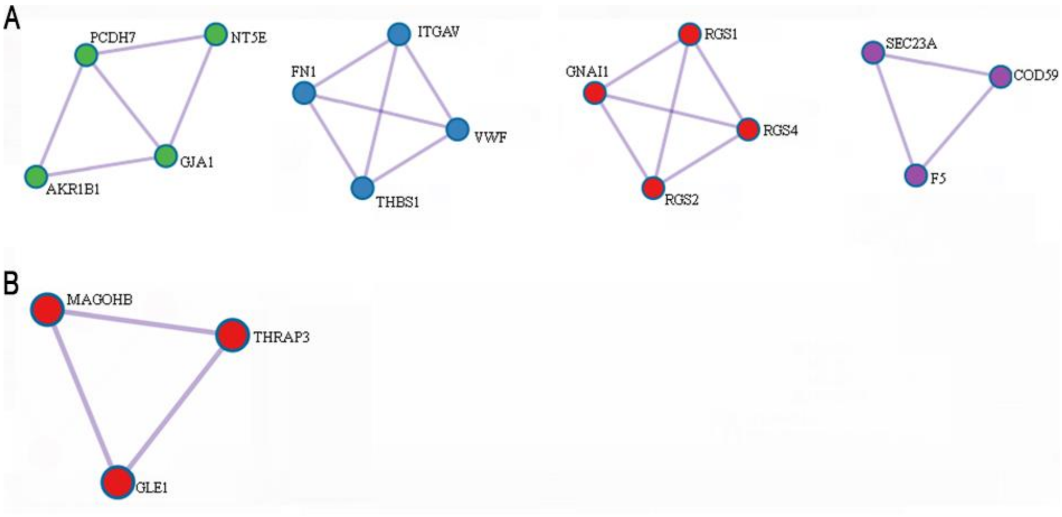


**Figure S1.** Protein-Protein Interaction Networks for Group A and Group B. A) Group A's PPI network. The Metascape MCODE algorithm was applied to cluster enriched ontology terms and identify densely connected protein neighborhoods. Each MCODE network is color-coded. B) Group B's PPI network.

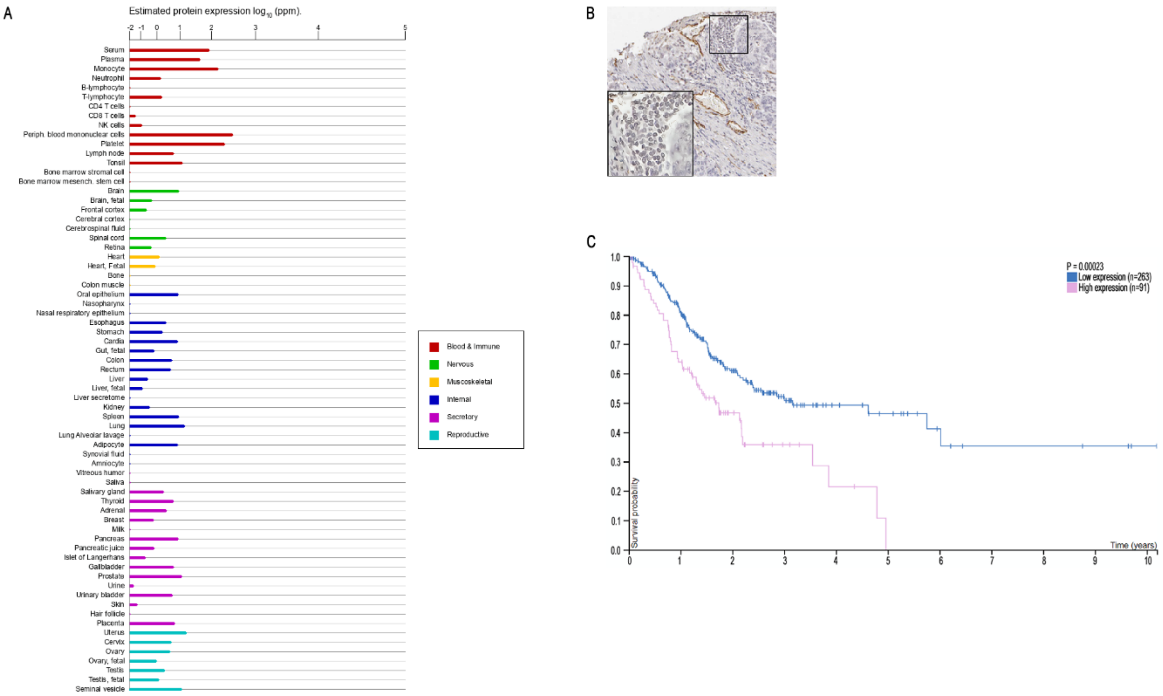


**Figure S2.** vWF Protein Expression, Staining, and Survival Analysis in Gastric Cancer. (A) vWF protein expression in normal tissues and various cell lines. (B) Immunohistochemical staining pattern of vWF protein in GC, showing negative expression in normal tissue. (C) Kaplan-Meier survival curve related to vWF expression in GC.

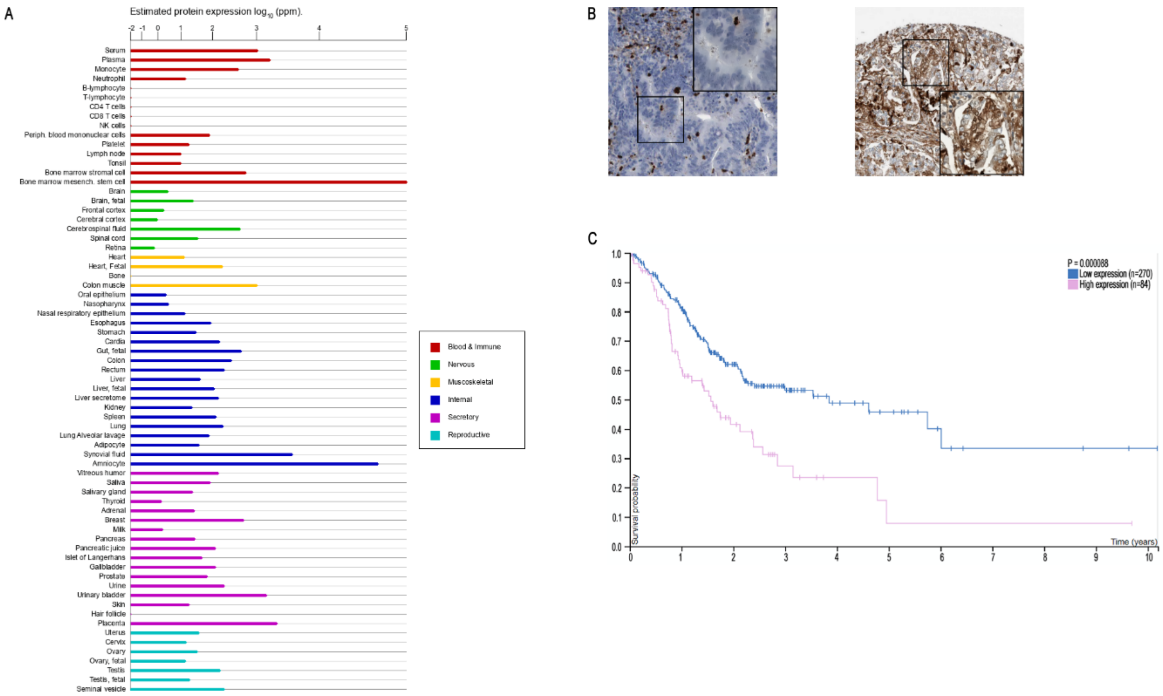


**Figure S3.** FN1 Protein Expression, Staining Patterns, and Survival Analysis in Gastric Cancer. (A) FN1 protein expression in normal tissues and various cell lines. (B) Immunohistochemical staining pattern of FN1 in normal tissue with negative staining. (C) Positive staining pattern of FN1 in cancer cells. (D) Kaplan-Meier survival curve related to FN1 expression in GC.

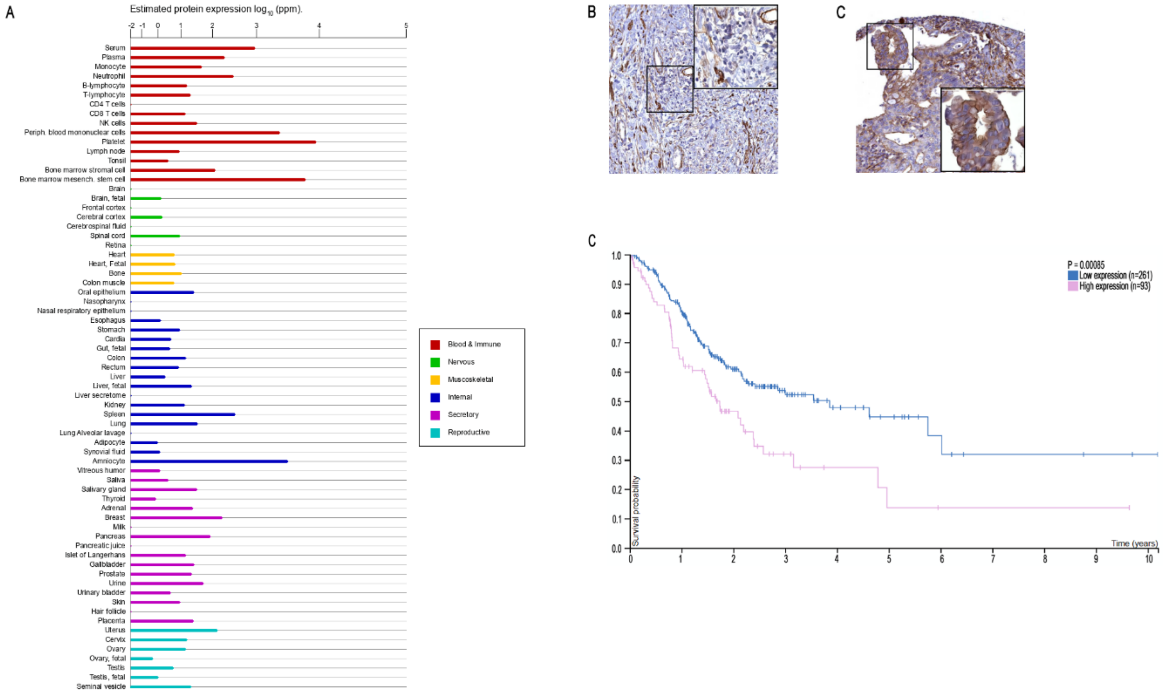

**Figure S4.** THBS1 Protein Expression, Staining Patterns, and Survival Analysis in Gastric Cancer. (A) THBS1 protein expression in normal tissues and various cell lines. (B) Immunohistochemical staining pattern of THBS1 in normal tissue with negative staining. (C) Positive staining pattern of THBS1 in cancer tissue. (D) Kaplan-Meier survival curve related to THBS1 expression in GC.

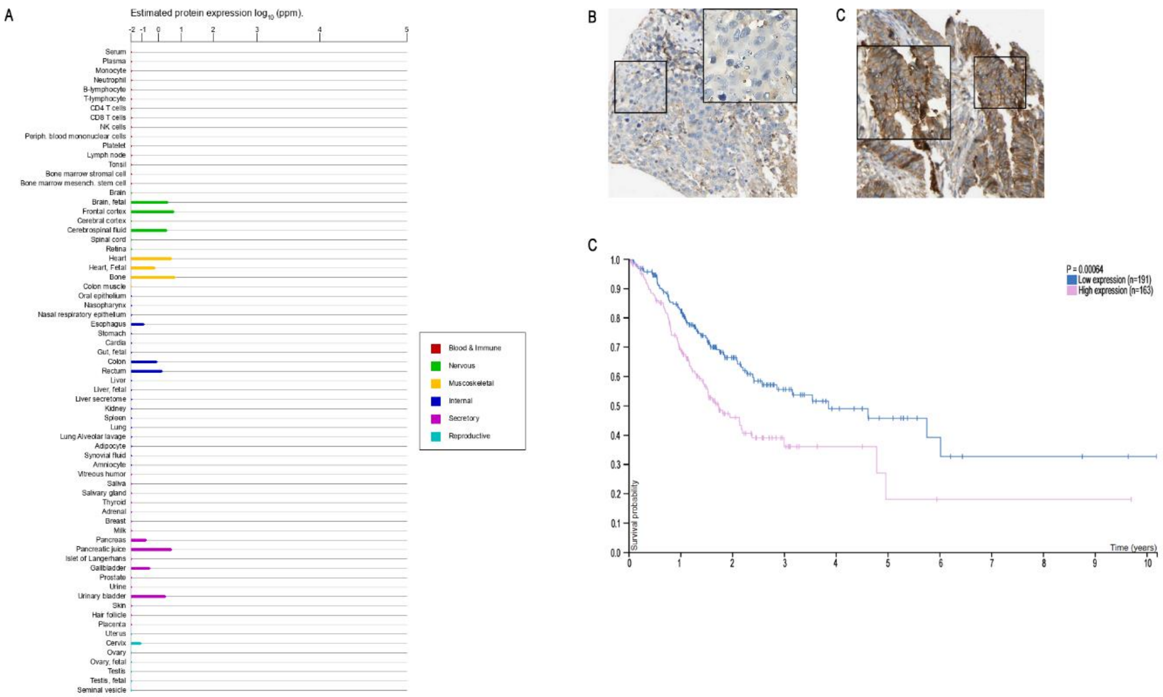


**Figure S5.** PCDH7 Protein Expression, Staining Patterns, and Survival Correlation in Gastric Cancer. A) Expression of PCDH7 proteins in normal tissues and various cell lines. B) and C) present the immunohistochemical staining pattern of PCDH7 protein related to GC, showing normal tissue with negative staining and positive labelling in cancer cells, respectively. D) Graph of GC survival related to PCDH7 expression.

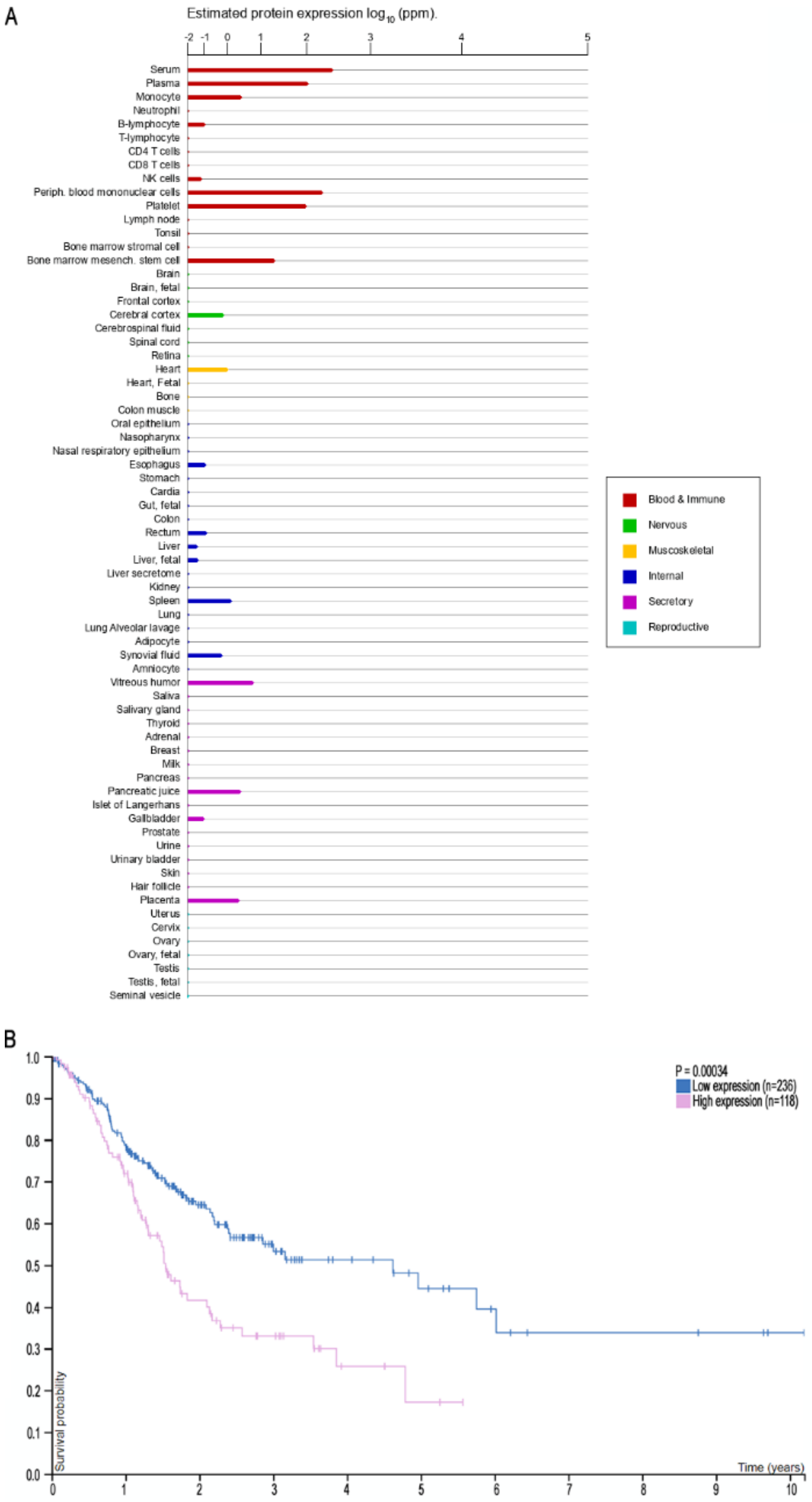


**Figure S6.** F5 Protein Expression and Survival Analysis in Gastric Cancer. (A) F5 protein expression in normal tissues and various cell lines. (B) Kaplan-Meier survival curve associated with F5 expression in GC.
