# Supplementary figures and images for: In Silico Insights Reveal Fibronectin 1 as a Theranostic Marker in Gastric Cancer
Source: Int J Mol Sci. 2024 Oct 16;25(20):11113. doi: 10.3390/ijms252011113 (PMC11507984; doi:10.3390/ijms252011113)

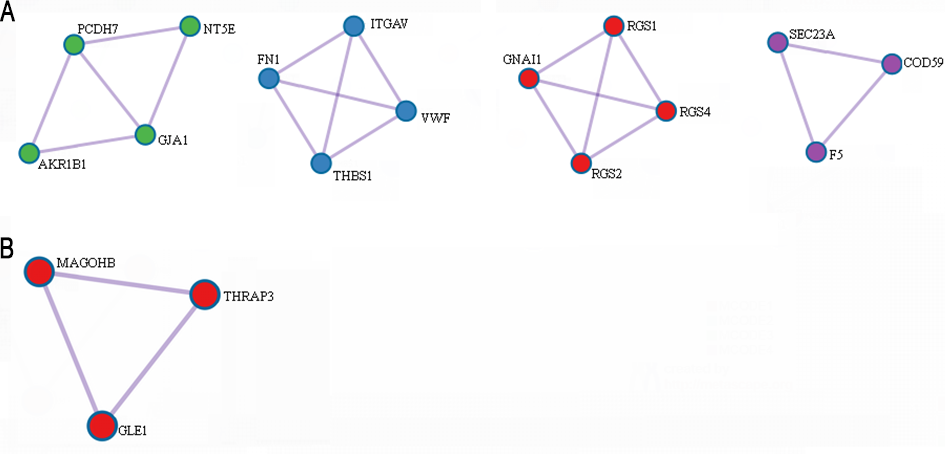

Supplement: Supplementary file 1 [file ijms-25-11113-s001.zip › Figure S1.tiff]

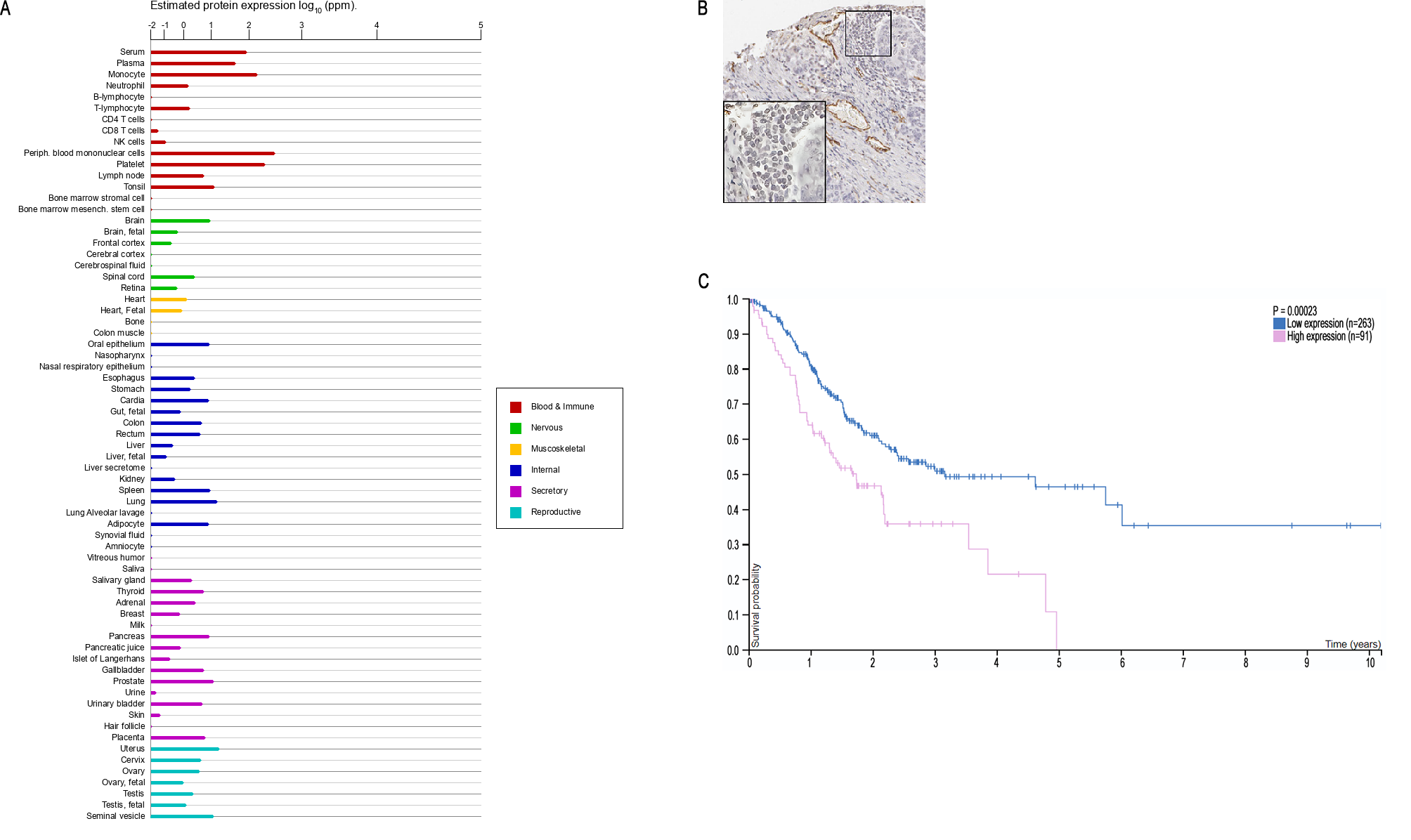

Supplement: Supplementary file 1 [file ijms-25-11113-s001.zip › Figure S2.tiff]

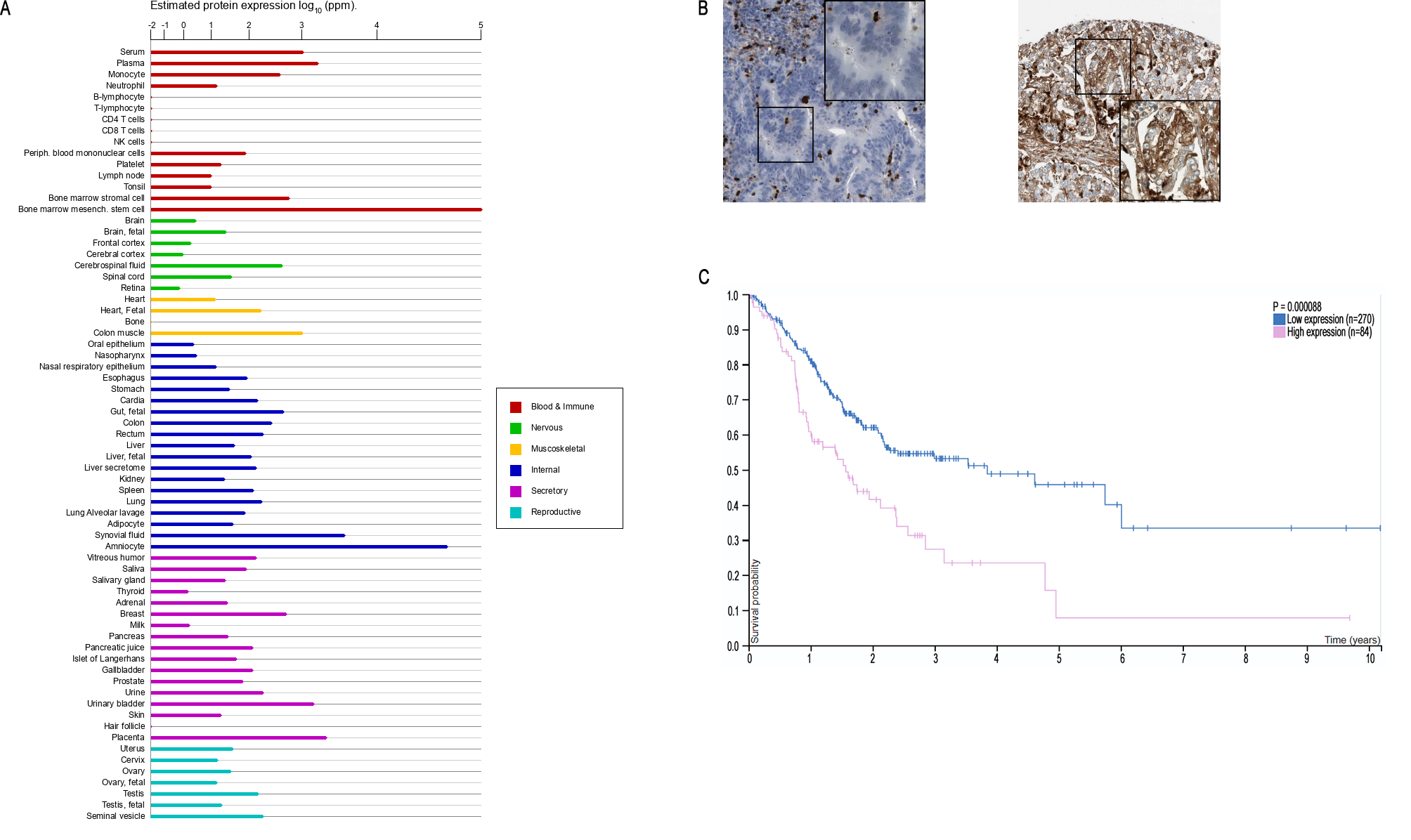

Supplement: Supplementary file 1 [file ijms-25-11113-s001.zip › Figure S3.tiff]

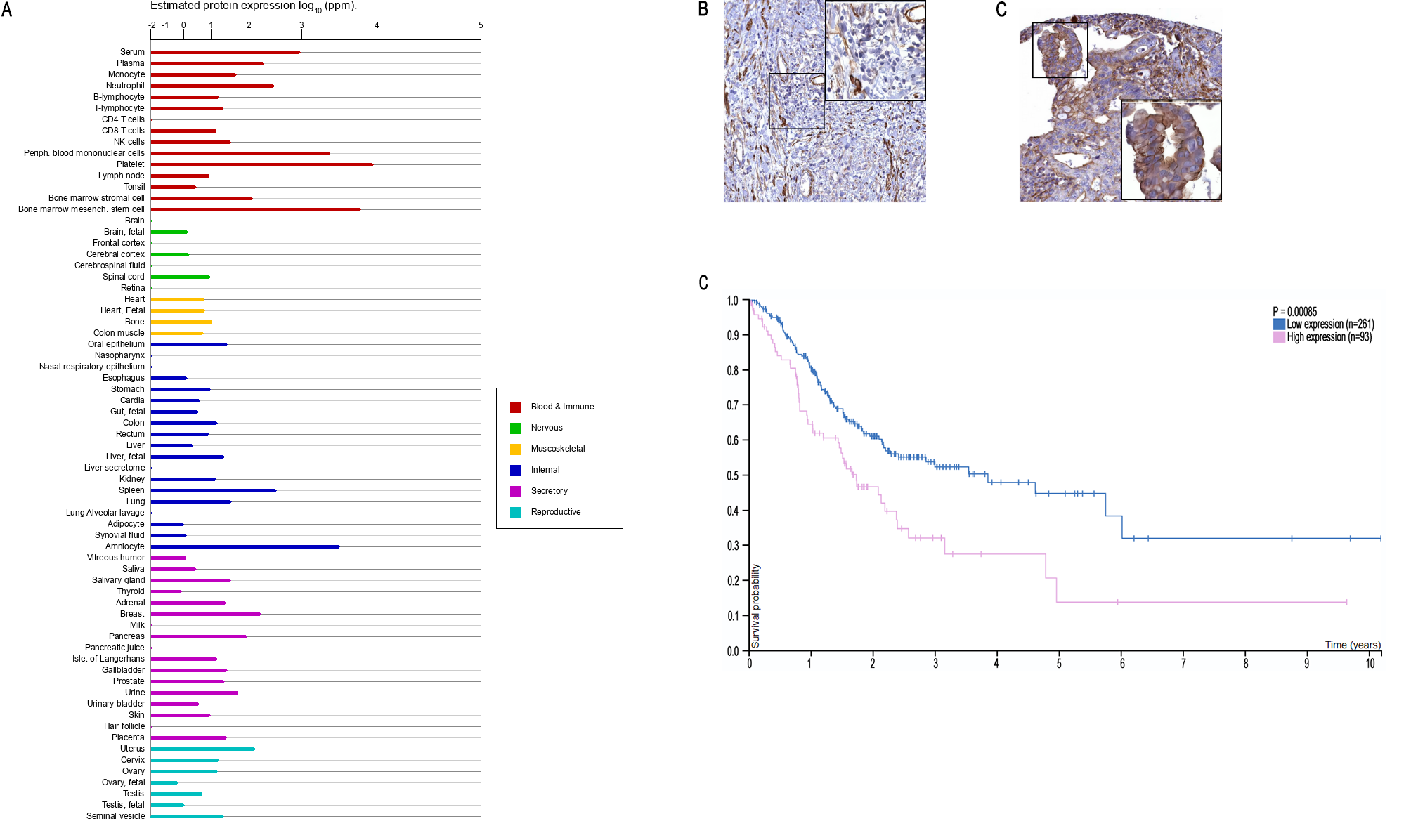

Supplement: Supplementary file 1 [file ijms-25-11113-s001.zip › Figure S4.tiff]

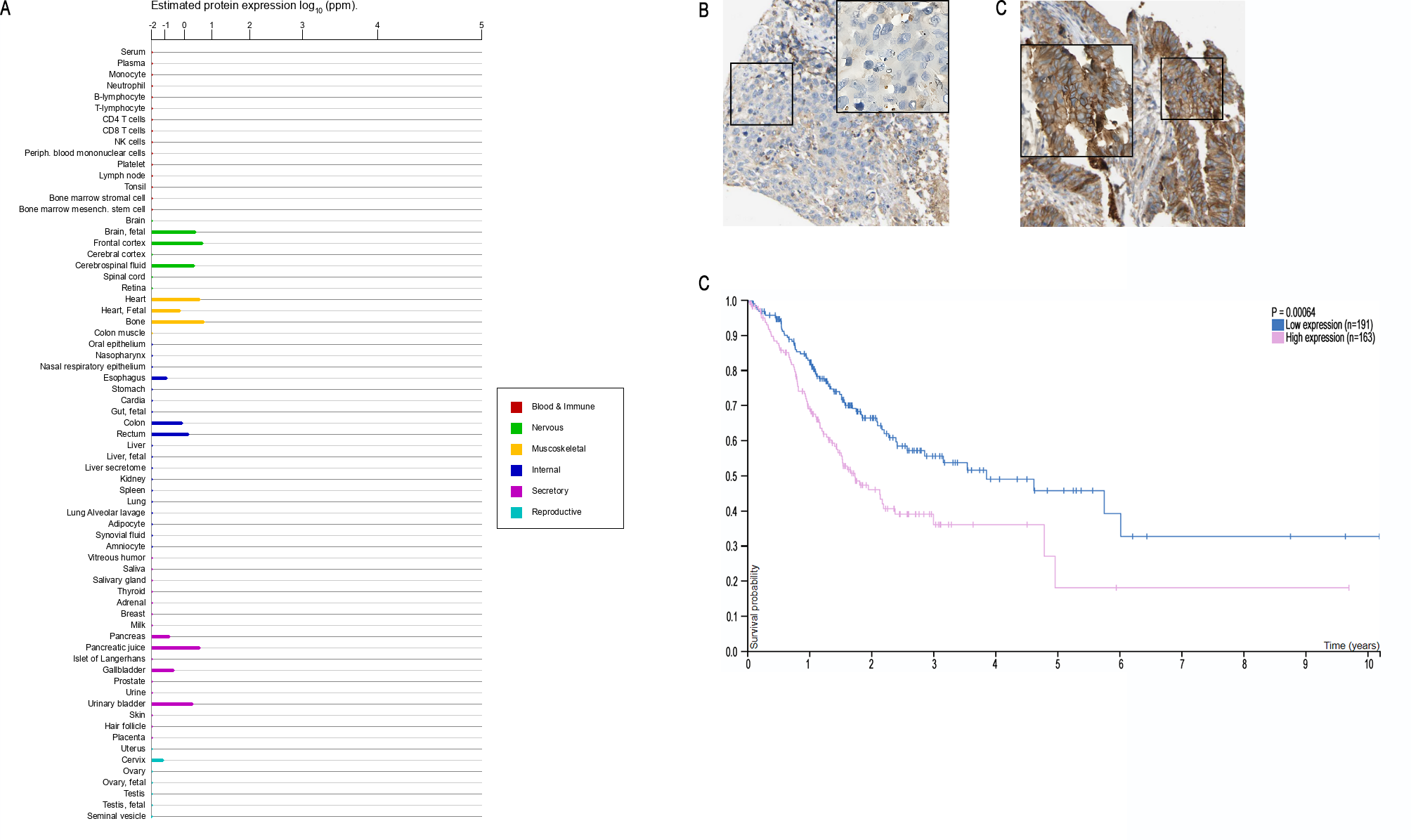

Supplement: Supplementary file 1 [file ijms-25-11113-s001.zip › Figure S5.tiff]

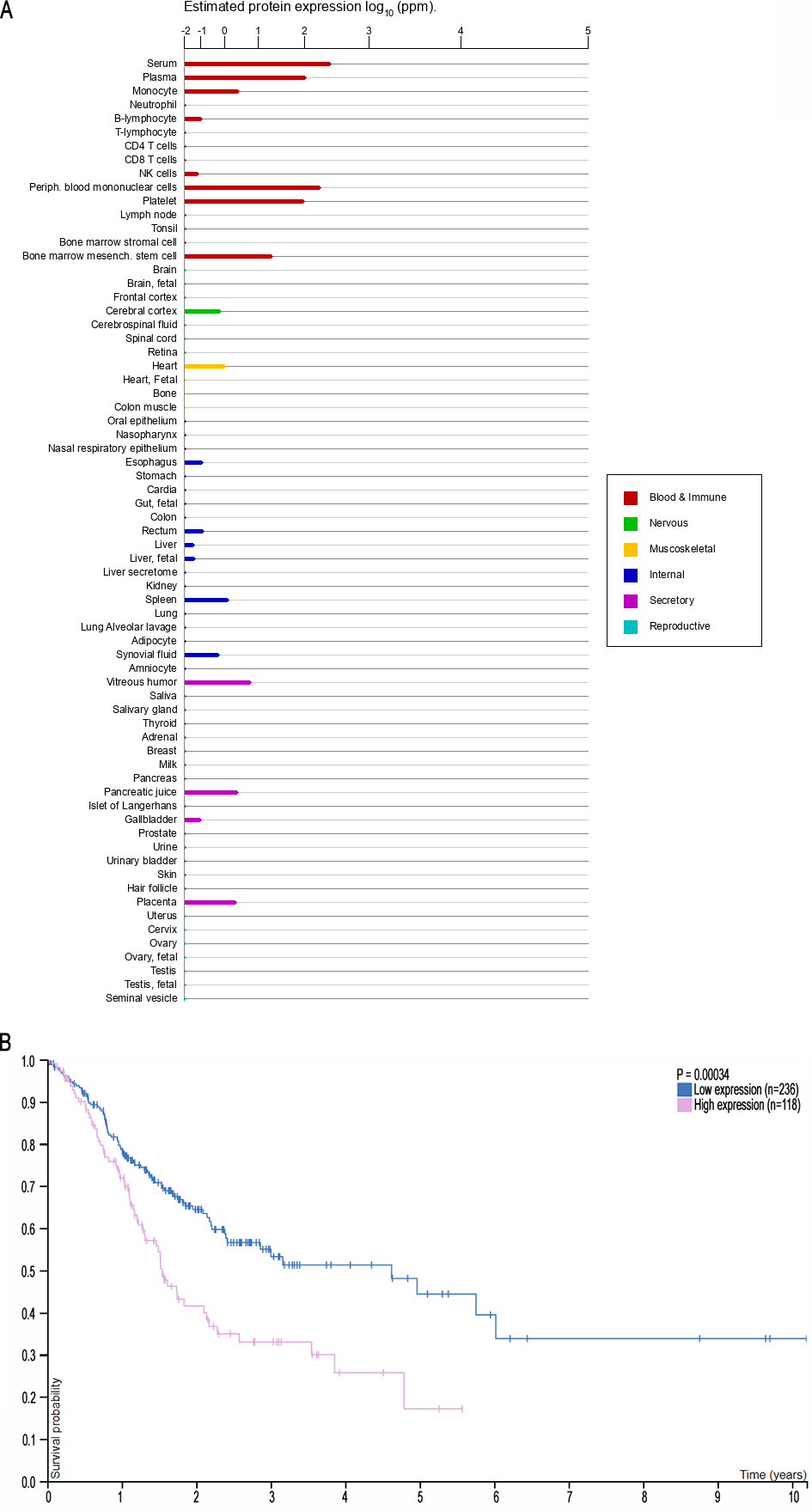

Supplement: Supplementary file 1 [file ijms-25-11113-s001.zip › Figure S6.tiff]
